# High-throughput screening identifies the activity of histone deacetylase inhibitors in patient-derived models of soft tissue sarcoma

**DOI:** 10.1080/15384047.2025.2589666

**Published:** 2025-11-30

**Authors:** Molly R. Danks, Piotr J. Manasterski, Henry Beetham, John C. Dawson, Richard J. R. Elliott, Jayne Culley, Rashi Krishna, Morwenna Muir, John P. Thomson, Ailsa J. Oswald, Ailith Ewing, William G. J. Kerrison, Paul H. Huang, Ioanna Nixon, Neil O. Carragher, Valerie G. Brunton

**Affiliations:** a Cancer Research UK Scotland Centre, Institute of Genetics and Cancer, University of Edinburgh, Edinburgh, UK; b MRC Human Genetics Unit, Institute of Genetics and Cancer, University of Edinburgh, Edinburgh, UK; c Division of Cancer Biology, The Institute of Cancer Research, Sutton, UK; d The Beatson West of Scotland Cancer Centre, Great Western Road, Glasgow, UK; e Department of Management Science, Strathclyde University, Glasgow, UK

**Keywords:** HDAC inhibitors, quisinostat, doxorubicin, undifferentiated pleomorphic sarcoma, leiomyosarcoma

## Abstract

**Background:**

Undifferentiated pleomorphic sarcoma (UPS) is a rare and aggressive soft tissue sarcoma with limited treatment options and a poor prognosis. As a complex karyotype tumor, UPS lacks recurrent targetable mutations, and response rates to standard first-line doxorubicin therapy are low. Phenotypic drug screening offers an alternative approach to identify new therapeutic targets without requiring prior knowledge of molecular mechanisms.

**Methods:**

A library of FDA-approved compounds and a custom histone deacetylase (HDAC) inhibitor library were screened using well-annotated patient-derived cell lines. Hit compounds were further characterized using apoptosis assays and *in vivo* xenograft studies. Biomarkers of activity were evaluated using gene expression and western blot analyses. Synergy with doxorubicin was evaluated in combination assays.

**Results:**

HDAC inhibitors emerged as a promising therapeutic class, demonstrating low IC_50_ values across cell lines (14.8−26.89 nM), with quisinostat taken forward for further evaluation. Gene expression changes in *EPAS1, FOXO1, AMOT,* and *FOSL1* were observed as potential biomarkers of activity. Combination assays revealed synergy between quisinostat and doxorubicin (average ZIP score: 1.02−15.65; ZIP_max_: 3.98−33.71), increasing apoptotic cell death *in vitro*. *In vivo*, quisinostat alone and in combination with doxorubicin significantly reduced the tumor volume (vehicle 160.0 ± 63.2 mm^3^, doxorubicin 78.0 ± 35.2 mm^3^, quisinostat 84.3 ± 13.1 mm^3^, and combination 49.2 ± 10.2 mm^3^). Quisinostat also showed potent activity in leiomyosarcoma (LMS) cell lines (5.82−31.32 nM), which represent an additional complex karyotype soft tissue sarcoma.

**Conclusions:**

Quisinostat demonstrated strong preclinical activity and synergy with standard-of-care doxorubicin in models of UPS and LMS.

## Introduction

Soft tissue sarcomas (STS) are a highly heterogeneous group of mesenchymal tumors accounting for approximately 1% of all adult cancers.^1^ Undifferentiated pleomorphic sarcoma (UPS), previously known as malignant fibrous histiocytoma (MFH), accounts for up to 20% of all cases and is a highly aggressive tumor characterized by atypical, pleomorphic spindle cells, lacking a specific line of differentiation. The standard treatment for localized UPS is surgery, although despite aggressive surgical resection, UPS has a high risk of recurrence and metastasis.^2^^,^^3^ In the advanced disease setting, treatment options are limited. Doxorubicin remains the preferred first-line therapy, although response rates are low.^4^^,^^5^

UPS is a complex karyotype tumor, and recent sequencing efforts have provided insights into its genetic heterogeneity,^6^^,^^7^ helping to identify potential new therapeutic approaches.^8^ However, as seen with efforts in other STS subtypes, routine sequencing in the clinical setting has not provided a step change in the treatment of sarcoma patients.^9^^,^^10^ An alternative approach to identifying potential new therapeutic targets is to employ a phenotypic drug screening pipeline, which does not require prior knowledge of a molecular target or its role in disease.^11^ Phenotypic screening can quickly test a range of drug classes, identifying compounds or target classes that kill cancer cells. This is particularly advantageous in diseases such as UPS with unmet therapeutic needs and where target biology is poorly understood.

Here, we describe a screening approach to identify potential new targets for UPS, which revealed sensitivity to histone deacetylase (HDAC) inhibitors in a range of *in vitro* models. HDAC1 and HDAC2 are upregulated in many solid tumors where their overexpression has been associated with poor prognosis;^12^ they remove acetyl groups from histone and non-histone proteins and are involved in epigenetic silencing of cell cycle and apoptosis regulators, allowing unchecked progression through the cell cycle and resistance to apoptosis.^12^^,^^13^ There is limited information on HDAC expression in STS,^14-17^ although at the mRNA level *HDAC1* and *HDAC2* are associated with shorter survival rates.^18^

Currently, several HDAC inhibitors are approved for the treatment of hematological malignancies.^12^^,^^13^^,^^19^ Although there are reports of partial responses and stable disease in Phase I/II trials in STS of mixed histotypes, either alone or in combination with chemotherapy,^20-26^ these have not been taken forward.

We show the sensitivity of well-annotated patient-derived UPS cell lines ^8^ to several HDAC inhibitors and identify a synergistic combination between quisinostat, a second-generation HDAC inhibitor, and doxorubicin. This synergy results in increased apoptotic cell death both *in vitro* and in mouse xenograft models. Furthermore, sensitivity to HDAC inhibitors was confirmed in a series of patient-derived leiomyosarcoma (LMS) cell lines. LMS represents an additional STS subtype with a complex karyotype for which there are currently few therapeutic options.^27^

## Results

### High-throughput screen

**Figure 1.**
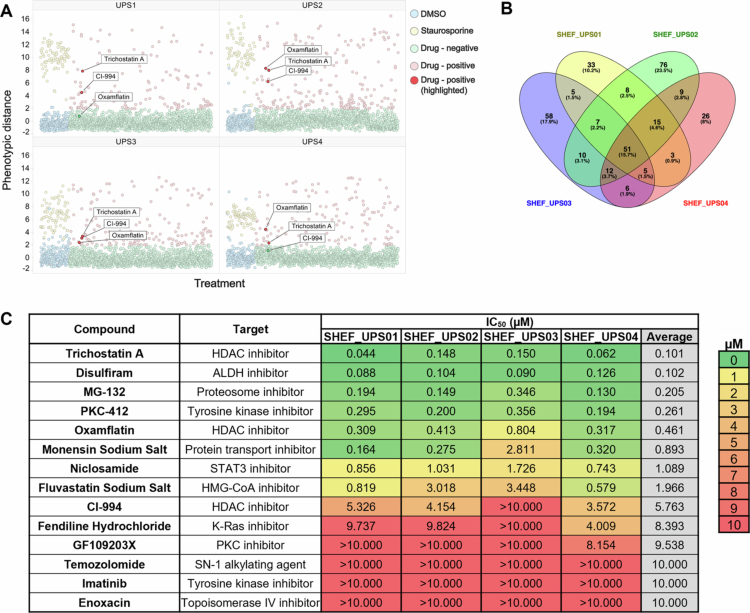
Summary of Prestwick FDA approved chemical library and SCREEN-WELL PKE library screens. Cell cycle and cell number analysis for 4 UPS cell lines. (A) Phenotypic distance per cell line for each compound; DMSO (negative) and staurosporine (positive) were used as controls. The HDAC inhibitors CI-994, oxamflatin and trichostatin A are shown as exemplars. (B) Number of hit compounds in each cell line created with Venny 2.1 (https://bioinfogp.cnb.csic.es/tools/venny/index.html). (C) Table of IC_50_ values for compounds taken forward for hit validation. IC_50_ values were calculated from dose‒response curves of normalized nuclei counts following 72-h treatment. *n* = 4 data points per dose per cell line (*n* = 2 biological repeats of technical duplicates).

Using cell number (nuclei count) and cell cycle phase as readouts, a total of 1,448 distinct compounds were tested at 10 µM across four UPS cell lines, which we previously confirmed to recapitulate the common genomic events observed in UPS patient tumors.^8^ The Euclidean distance (phenotypic distance) was calculated relative to the centroid of the DMSO controls. A phenotypic distance with a z-score greater than 3 was considered a hit compound. We identified 324 compounds (22%) as hits in at least 1 cell line, with 51 (4%) active in all four cell lines (Figure 1A, B). Hit compounds were triaged by excluding toxic compounds (with fewer than 10 DNA structures per well), those withdrawn from clinical use and common anti-cancer agents that have historically been extensively investigated across pan-cancer indications. This led to 14 compounds being taken forward for validation (Figure 1C and Supplementary Figure S1).

The 14 selected compounds were tested in an eight-point half-log dose‒response ranging from 10 µM to 3 nM across the panel of four UPS cell lines used in the original screen (Supplementary Figure S2). Six of the 14 (42.9%) compounds selected from the original screens passed validation, defined as compounds with an IC_50_ < 1 µM across ≥3 cell lines (Figure 1C). The HDAC inhibitor trichostatin A was the most potent hit, with an IC_50_ ≤ 150 nM for all the tested cell lines (Figure 1C). Among the top 5 compounds, another HDAC inhibitor, oxamflatin, had an average IC_50_ value of 461 nM across the cell lines (Figure 1C). Trichostatin A showed a greater response in SHEF_UPS01 (IC_50_ = 44 nM) and SHEF_UPS04 (IC_50_ = 62 nM), with SHEF_UPS02 (IC_50_ = 148 nM) and SHEF_UPS03 (IC_50_ = 150 nM) exhibiting IC_50_ values more than twice those observed in the other cell lines. Similar to trichostatin A, SHEF_UPS03 exhibited an IC_50_ value (804 nM) nearly double that of the other cell lines (309−413 nM). A third HDAC inhibitor was also included in validation, CI-994, although this compound did not pass validation, with IC_50_ values ranging from 3.572 µM to >10 µM.

### HDAC inhibitor screen

**Figure 2.**
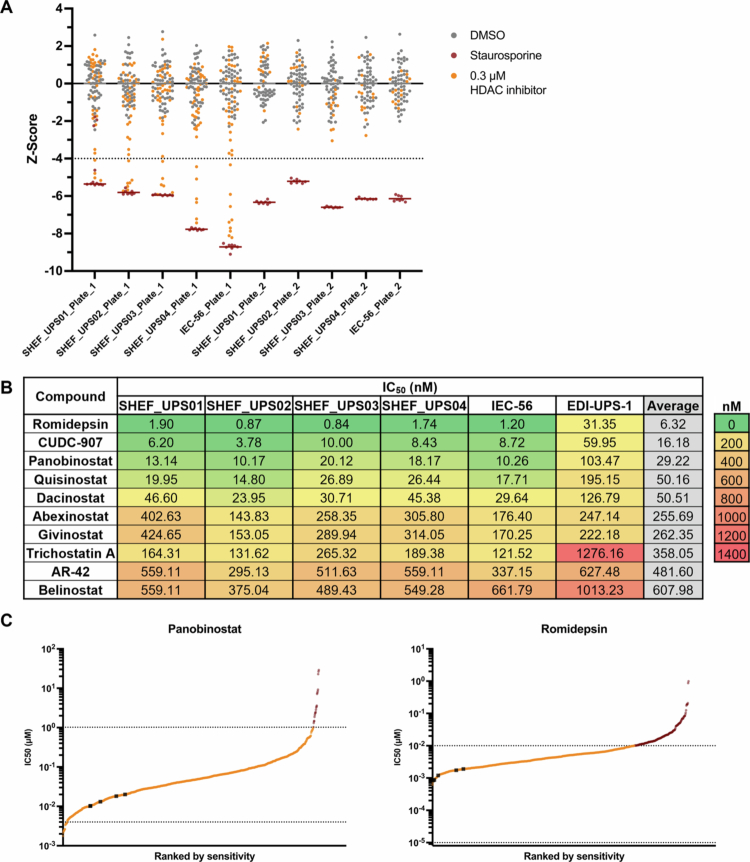
Custom HDAC inhibitor screen and validation in UPS cell lines. (A) Univariate analysis of normalized z-scores (nuclei counts) across each plate. Z-score < – 4 threshold for hit selection. The gray (DMSO) and red (staurosporine) lines represent the means of the controls, and all HDAC inhibitors are shown at 0.3  μM. (B) Table of IC_50_ values from hit HDAC inhibitor validation. IC_50_ values were calculated from dose‒response curves of normalized nuclei counts following 72-h treatment. *n* = 8 data points per dose per cell line (*n* = 2 biological repeats of technical duplicates). (C) Pan-cancer IC_50_ values of HDAC inhibitors ranked by sensitivity; black squares are the UPS cell line IC_50_ values from the HDAC inhibitor screen validation, and red circles are IC_50_ values in the GDSC database above the maximum tested concentration. The upper and lower dotted lines represent the highest and lowest concentrations tested in the database, respectively. Data from The Genomics of Drug Sensitivity in Cancer database.^29^

As we had identified sensitivity to HDAC inhibitors across the cell lines, we then screened a custom HDAC inhibitor library (Supplementary Table S1) consisting of 53 compounds. Each compound was tested in an eight-point half-log single replicate dose‒response ranging from 10 µM to 3 nM across a panel of five UPS cell lines, including the four previously used and a novel PDX-derived cell line we recently generated.^8^ Hits were defined as compounds that had a z-score less than −4 in 3 or more cell lines at 0.3 µM (Figure 2A). Z-scores were calculated from univariate analysis of nuclei counts normalized to those of DMSO controls. As the screen was carried out in a single replicate, all hit compounds were cross-checked against dose‒response curves (Supplementary Figure S3) to ensure low AUC values. Ten hit compounds were identified based on z-scores: eight pan-HDAC inhibitors (AR-42, belinostat, panobinostat, and trichostatin A), including three which are more potent inhibitors of HDAC1 (abexinostat, dacinostat, and quisinostat), and one more potent inhibitor of HDAC1 and HDAC2 (givinostat); one selective HDAC1 and HDAC2 inhibitor (romidepsin); and one dual HDAC (class I and II) and phosphoinositide 3-kinase (PI3K, class Iα, *β* and *δ*) inhibitor (CUDC−907/fimepinostat).

These ten compounds were then reassessed, and IC_50_ values were calculated from eight-point half-log dose‒response curves in the five UPS cell lines and low-passage cells derived from a primary UPS (Figure 2B and Supplementary Figure S4A). Overall, the responses to each treatment were similar among the established cell lines. SHEF_UPS02 exhibited the highest sensitivity to compounds compared to the other four cell lines, with the lowest IC_50_ value for eight out of ten compounds. Romidepsin (HDAC1 and HDAC2 inhibitor) was the most potent compound, with an average IC_50_ of 1.31 nM across established cell lines, with SHEF_UPS02 (0.87 nM) and SHEF_UPS03 (0.84 nM) exhibiting subnanomolar IC_50_ values. CUDC-907 (dual HDAC and PI3K inhibitor), panobinostat (pan-HDAC inhibitor), quisinostat (pan-HDAC, potent HDAC1 inhibitor) and dacinostat (pan-HDAC, potent HDAC1 inhibitor) were also efficacious, with IC_50_ values less than 50 nM across established cell lines. Patient-derived low-passage EDI-UPS-1 cells consistently exhibited higher IC_50_ values – at least 5-fold greater – for the top-performing compounds (romidepsin, CUDC-907, panobinostat, and quisinostat), although the relative sensitivity patterns among the compounds remained similar. For example, romidepsin remained the most potent compound in EDI-UPS-1, with an IC_50_ of 31.35 nM. In contrast, the IC_50_ values for compounds with lower potency (>100 nM) were similar between EDI-UPS01 and established UPS cell lines.

**Figure 3.**
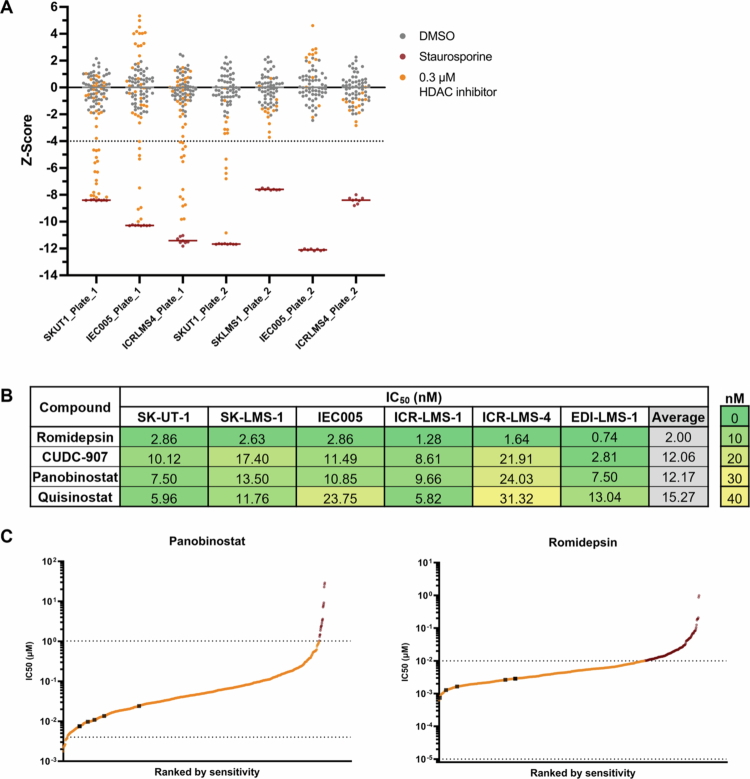
Custom HDAC inhibitor screen and validation in LMS cell lines. (A) Univariate analysis of normalized z-scores (nuclei counts) across each plate. Z-score < –4 threshold for hit selection. The gray (DMSO) and red (staurosporine) lines represent the means of the controls, and all HDAC inhibitors are shown at 0.3 μM. (B) IC_50_ values across cell lines for HDAC inhibitors. IC_50_ values calculated from dose‒response curves of normalized nuclei counts following 72-h treatment. *n* = 9 data points per dose per cell line (*n* = 3 biological repeats of technical triplicates). (C) Pan-cancer IC_50_ values of HDAC inhibitors ranked by sensitivity; black squares are the LMS cell line IC_50_ values from the HDAC inhibitor screen validation, and red circles are IC_50_ values in the GDSC database above the maximum tested concentration. The upper and lower dotted lines represent the highest and lowest concentrations tested in the database, respectively. Data from The Genomics of Drug Sensitivity in Cancer database.^29^

As the upregulation of class I HDACs has been reported in leiomyosarcoma (LMS)^17^ and computational analyses have identified HDACs as potential synthetic lethal targets in LMS,^28^ we then screened the HDAC inhibitor library across a panel of four LMS cell lines (Figure 3A). Notably, 9 of the top 10 compounds overlapped between the UPS and LMS cell lines in the screen. Following validation in the UPS cell lines, the top four HDAC inhibitors were further tested in five LMS cell lines and low-passage cells derived from a primary LMS (Figure 3B and Supplementary Figure S4B). The ranking of these four compounds was consistent across both subtypes, and the average response to each compound was comparable between UPS and LMS models. In contrast to EDI-UPS-1, the primary low-passage LMS cells, EDI-LMS-1, were the most sensitive overall, showing the lowest IC_50_ values for romidepsin (0.74 nM), panobinostat (7.50 nM), and CUDC-907 (2.81 nM). SK-UT-1 and ICR-LMS-1 exhibited similar responses across all compounds. Similarly, SK-LMS-1 and IEC005 showed comparable sensitivity, except for quisinostat, where IEC005 was twice as resistant (IC_50_: 23.75 nM) as SK-LMS-1 (11.76 nM). ICR-LMS-4 was the most resistant cell line, with the highest IC_50_ values for CUDC-907 (21.91 nM), panobinostat (24.03 nM) and quisinostat (31.32 nM).

### Sarcomas are sensitive to HDAC inhibitors

As the UPS and LMS cell lines were sensitive to class I HDAC inhibitors, IC_50_ values were compared to those in The Genomics of Drug Sensitivity in Cancer (GDSC) database for other STS and cancer types.^29^ Importantly, the GDSC data are based on a fluorescence-based viability assay, while this study measured nuclei counts, which may affect direct comparisons of IC_50_ values. Data were available for six compounds: romidepsin, panobinostat, AR-42, belinostat, dacinostat, and trichostatin A. IC_50_ values were available for at least 718 cell lines per compound across 13 tissue types, including 16–20 STS cell lines, including rhabdomyosarcoma, liposarcoma, LMS, synovial sarcoma, fibrosarcoma, fibrous histiocytoma, malignant fibrous histiocytoma, and epithelioid sarcoma. Ranked sensitivity plots showed that the UPS and LMS cell lines were within the lower half of the sensitive cell lines across all the compounds (Figures 2C and 3C, and Supplementary Figure S5 for all the available compounds). The comparison with the GDSC database confirms that HDAC inhibitors are potent drugs across both UPS and LMS cell lines.

### Genomic analysis of HDAC gene alterations

HDAC genes are infrequently mutated in STS, with most genomic events affecting these genes occurring at the copy number level rather than through single nucleotide variants.^6^^,^^8^ To investigate whether such alterations might underlie the observed sensitivity to HDAC inhibition, we analyzed genomic data on the patient-derived UPS cell lines ^8^ and integrated this with analysis of publicly available and in-house datasets from solid UPS and LMS tumors, including data from the Pan-Cancer Analysis of Whole Genomes (PCAWG) project and Genomics England (GEL) (Supplementary Figure S6).^8^^,^^9^^,^^30^

Across these datasets, copy number loss involving one or more HDAC genes, particularly class I members such as *HDAC1*, was a frequent event in all solid tumor samples. High-level copy number gain or amplification of HDAC genes was comparatively rare (Supplementary Figure S6). Despite differences in HDAC gene copy number changes across our cell line models, there was no correlation between these genomic events and the sensitivity to HDAC inhibition. These findings suggest that while HDAC genes are recurrently altered at the copy number level in UPS, LMS and other solid tumors, such alterations are not sufficient to predict sensitivity to HDAC inhibition. This underscores the importance of functional approaches such as phenotypic drug screening to uncover therapeutic vulnerabilities in STS with complex and variable genomic landscapes.

### Combination studies with HDAC inhibitors and doxorubicin

The four hit compounds – romidepsin, CUDC-907, panobinostat and quisinostat – were tested in combination with doxorubicin to explore their synergistic potential. Assays were carried out in a 6 (doxorubicin) × 5 (HDAC inhibitor) dose‒response matrix across all UPS cell lines. The average synergy scores for all the mathematical models (zero interaction potency (ZIP), highest single agent (HSA), Bliss and Loewe) are shown in Supplementary Figure S7A, and the full synergy matrices for doxorubicin and quisinostat are shown in Supplementary Figure S7B. A synergy score greater than 10 suggests that the interaction between two drugs is likely synergistic;^31^ scores from −10 to 10 suggest additive interactions, and scores less than −10 are considered antagonistic interactions.

**Figure 4.**
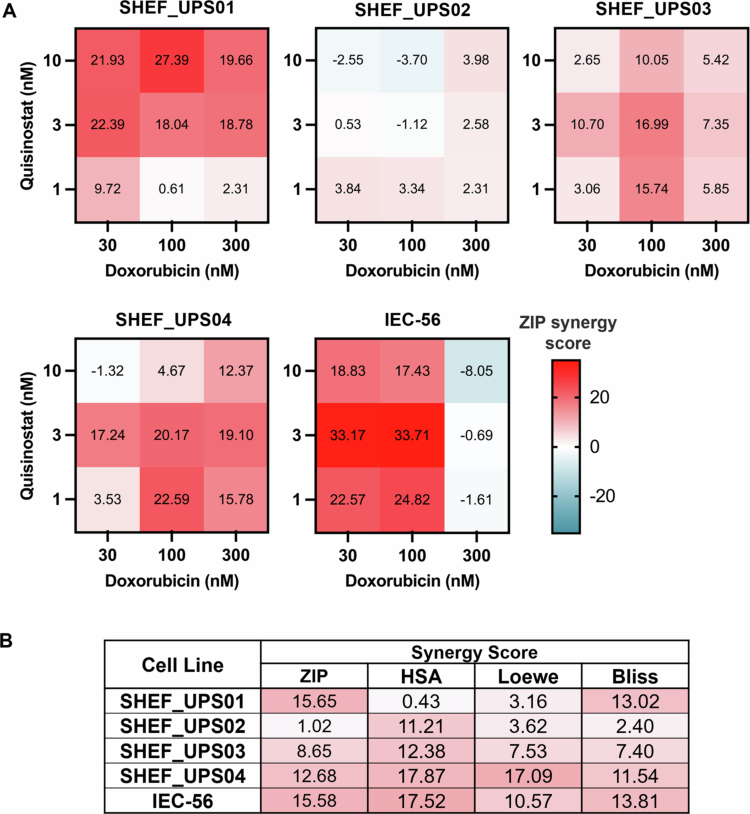
Synergy analysis of quisinostat in combination with doxorubicin. (A) ZIP synergy scores for selected doses across the UPS cell lines. (B) Average synergy scores for the selected doses across all mathematical models provided on synergyfinderplus.org. For full synergy plots and synergy scores, see Supplementary Figure S7. *n* = 6 data points per dose per cell line (*n* = 2 biological repeats of technical triplicates).

Synergistic interactions are limited across cell lines treated with doxorubicin and CUDC-907, panobinostat or romidepsin (Supplementary Figure S7). SHEF_UPS01 exhibited potential synergy when treated with four different combinations of low-dose CUDC-907 and doxorubicin (ZIP_max_ 21.12); only one potentially synergistic combination was observed in SHEF_UPS03 (ZIP_max_ 11.96), and none were observed across the other cell lines. Two or three synergistic combinations were observed in SHEF_UPS01-04 when treated with doxorubicin and panobinostat (ZIP_max_: 13.42−13.81), and none were observed in IEC-56. No potentially synergistic combinations were observed when SHEF_UPS02 cells were treated with romidepsin in combination with doxorubicin, whilst the other four cell lines elicited one to two potentially synergistic dose combinations. The greatest synergy was observed with doxorubicin in combination with low doses of quisinostat (Figure 4A). Despite all the cell lines exhibiting similar IC_50_ values in 2D assays when treated with quisinostat (14.8−26.89 nM) or doxorubicin (5−22 nM), the response to the combination was variable. The highest ZIP_max_ values were observed in SHEF_UPS01 (27.39) and IEC-56 (33.71) cells treated with 100 nM doxorubicin and 10 nM or 3 nM quisinostat, respectively. Similarly, dose combinations with 100  nM doxorubicin elicited a synergistic ZIP_max_ score in SHEF_UPS03 (16.99 at 3 nM quisinostat) and SHEF_UPS04 (22.59 at 1 nM quisinostat). SHEF_UPS02 did not exhibit any synergistic scores across the selected doses of interest. The lack of synergy in SHEF_UPS02 may reflect a predominantly cytostatic response to quisinostat driven by cell cycle arrest mechanisms rather than apoptosis, thereby limiting the synergistic effects observed in other models. Within this range of doses, the average ZIP score (Figure 4B) was greater than 10 for SHEF_UPS01 (15.65), SHEF_UPS04 (12.68), and IEC-56 (15.58). Across LMS models, quisinostat demonstrated synergistic potential with doxorubicin. Synergy was observed at low concentrations of both quisinostat (1−3 nM) and doxorubicin (10−30 nM), with near-synergistic ZIP_max_ values in SK-UT-1 (9.81) and synergistic ZIP_max_ values in all other cell lines (range: 12.34−15.66) (Supplementary Figure S8). These findings demonstrate that low-dose quisinostat enhances doxorubicin activity in LMS cell lines, which is consistent with the synergistic effects observed in UPS. As quisinostat showed the greatest synergistic potential with doxorubicin and exhibited low IC_50_ values in all the cell lines, it was prioritized for further validation in the SHEF_UPS01 and IEC-56 cell lines, which presented the greatest ZIP_max_ and average synergy scores.

### Effect of quisinostat and doxorubicin on cell death and proliferation pathways

**Figure 5.**
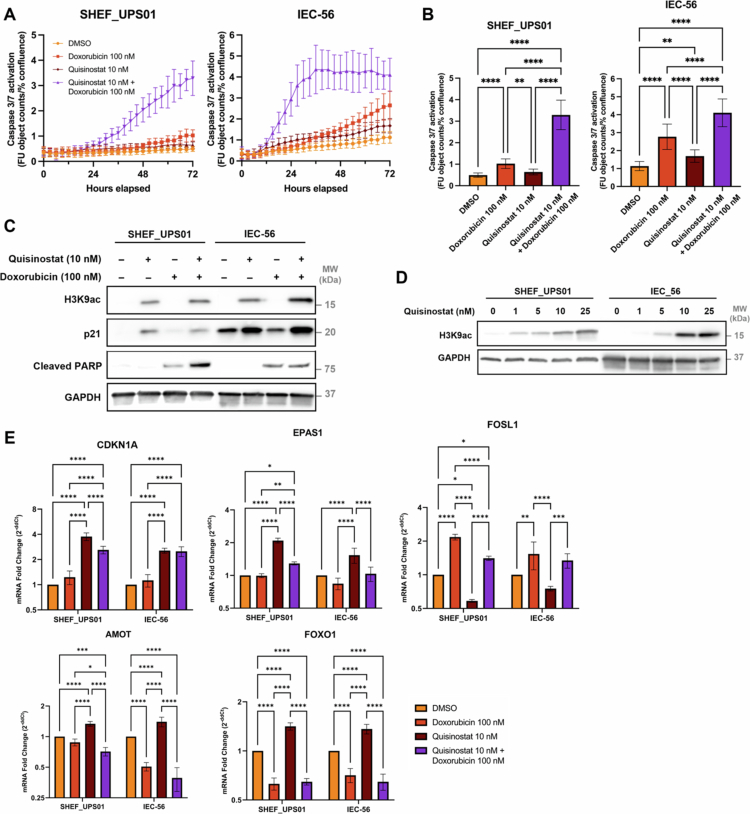
Effect of quisinostat and doxorubicin on cell death and proliferation pathways. (A, B) Effects of quisinostat, doxorubicin or their combination on caspase 3 activation in UPS cells (one-way ANOVA with Tukey’s multiple comparison test). *n* = 18 data points per treatment per cell line (*n* = 6 biological repeats of technical triplicates) (C, D) Western blot analysis of H3K9ac, p21, and cleaved PARP proteins after 48 h of treatment. (E) qPCR analysis of *CDKN1A*, *EPAS1*, *FOSL1*, *AMOT,* and *FOXO1* expression after 24 h of treatment (two-way ANOVA with Tukey’s multiple comparisons test). *n* = 4 data points per dose per cell line (*n* = 2 biological repeats of technical duplicates). For all, the data are presented as the mean ±  standard deviation. *p < 0.05, **p < 0.01, ***p < 0.001, ****p < 0.0001.

We then examined whether quisinostat and doxorubicin induced apoptosis in the UPS cell lines using a Caspase 3/7 activation assay. After 72 h of treatment, the combination of quisinostat and doxorubicin significantly increased the number of apoptotic cells compared to DMSO control and single-agent treatments in both SHEF_UPS01 and IEC-56 (Figure 5A, B). Quisinostat alone also significantly increased cell death in IEC-56, while doxorubicin alone was effective in both lines. This was accompanied by the induction of cleaved PARP (Figure 5C). An increase in H3K9ac in quisinostat-treated cells confirmed its on-target activity (Figure 5C, D). Consistent with the known effect of quisinostat on cell cycle arrest,^32^ there was an increase in p21 either alone or in combination with doxorubicin (Figure 5C), which was also observed at the mRNA level (*CDKN1A*, Figure 5E). Quisinostat also upregulated *EPAS1*, *FOXO1*, and *AMOT* (Figure 5E), all of which have been linked to the regulation of proliferation and survival in STS.^33-35^ *FOSL1* expression, which drives proliferation in UPS cell lines,^16^ significantly decreased in SHEF_UPS01 but showed no significant change in IEC-56 cells after quisinostat treatment (Figure 5E). In LMS cell lines (SK-UT-1 and IEC005), quisinostat treatment led to significant upregulation of *CDKN1A*, *EPAS1*, and *FOXO1* compared to other treatment groups, as well as a decrease in *FOSL1* expression, while *AMOT* expression remained unchanged (Supplementary Figure S9). The consistent changes in gene expression across UPS and LMS support further studies into potential biomarkers of quisinostat activity.

### Combined quisinostat and doxorubicin treatment reduces*in vivo* tumor growth

**Figure 6.**
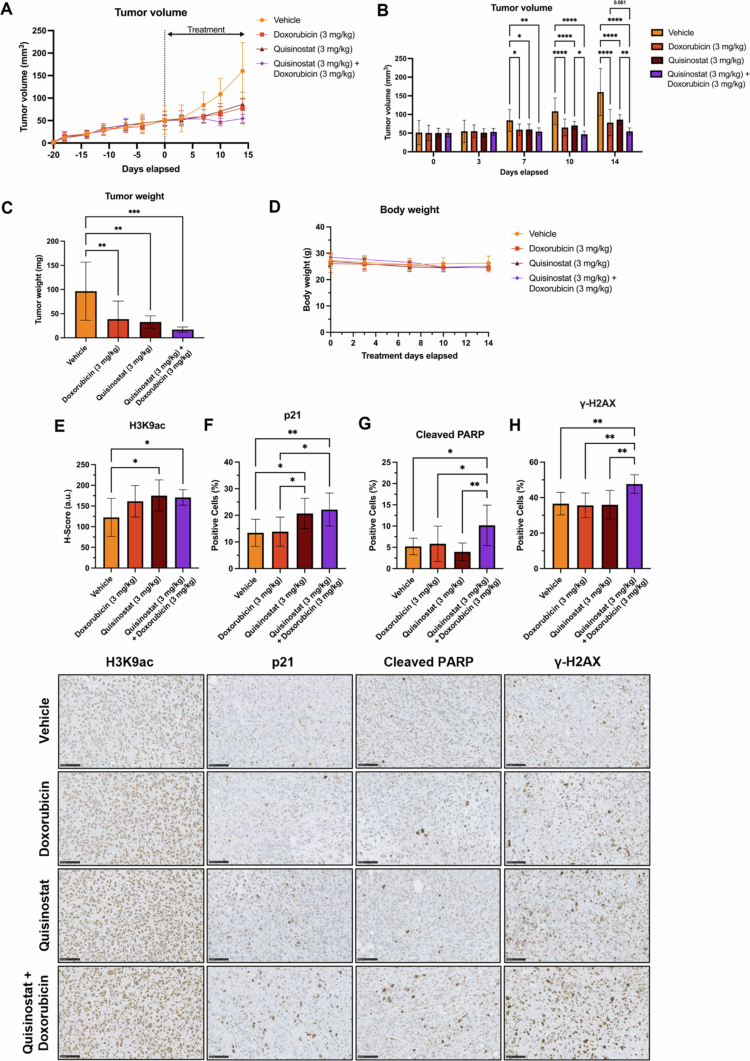
Anti-tumor effects of quisinostat plus doxorubicin in the IEC-56 cell-derived xenograft model. (A, B) Biweekly tumor volume (two-way ANOVA with Tukey’s multiple comparisons test) of mice bilaterally implanted subcutaneously with IEC-56 cells; the mice were randomized into four groups and treated with intraperitoneal injections for 14 d. (C) Final tumor weight (one-way ANOVA with Tukey’s multiple comparison test). *n* = 10 tumors per group. (D) Biweekly body weight measurements, *n* = 5 mice per group. (E-H) Representative images and quantification (one-way ANOVA with Tukey’s multiple comparison test) of treated tumors stained for (E) H3K9ac, (F) p21, (G) cleaved PARP and (H) *γ*-H2AX, *n* = 10 (5 tumors per group, 2 technical replicates each). Scale bars = 100 μm. For all, the data are presented as the mean ± standard deviation. *p < 0.05, **p < 0.01, ***p < 0.001, ****p < 0.0001.

The anti-tumor effect of quisinostat alone or in combination with doxorubicin was investigated in the IEC-56 cell-derived xenograft model. Following the establishment of tumors, the mice were treated with 3 mg/kg doxorubicin weekly, 3 mg/kg quisinostat daily or a combination of both compounds for 14 d. Significant differences in tumor volume between the control and treatment groups were observed from day 7 of treatment (Figure 6A, B and Supplementary Figure S10). Compared with the control group, at the end of the treatment period, all the treatment groups presented significantly reduced tumor volume; average volume: vehicle 160.0 ± 63.2 mm^3^, doxorubicin 78.0 ± 35.2 mm^3^, quisinostat 84.3 ± 13.1 mm^3^, and combination 49.2 ± 10.2 mm^3^. The combination treatment induced a significant reduction in tumor volume compared to quisinostat as a single agent and a non-significant reduction compared to doxorubicin (*p* = 0.061). All treatment groups induced a significant decrease in tumor weight compared to the control group (Figure 6C); average mass: vehicle 96.4 ± 60.1 mg, doxorubicin 38.5 ± 37.6 mg, quisinostat 32.7 ± 12.8 mg and combination 17.0 ± 5.5 mg. The average tumor weight in the combination group was lower than that of single agents, although not significantly different. There was no difference in body weight across the treatment groups (Figure 6D).

Quisinostat treatment, alone or in combination with doxorubicin, significantly increased H3K9ac expression compared to vehicle control, indicating the inhibition of HDAC activity (Figure 6E). A significant increase in p21-positive cells was observed in quisinostat- and combination-treated tumors compared to both vehicle- and doxorubicin-treated tumors (Figure 6F). Neither single agent induced a marked increase in cleaved PARP expression, but the combination significantly increased the percentage of cleaved PARP-positive cells compared with vehicle or single agents (Figure 6G). *γ*-H2AX levels were similar between the vehicle and single agent treatments but significantly increased with the combination (Figure 6H).

## Discussion

To identify potential new treatments for patients with UPS and LMS, we applied a phenotypic screening approach using well-annotated patient-derived cell lines, followed by validation in freshly dissociated tumor samples from both subtypes. These patient-derived models more accurately recapitulate the original tumors than established cell lines do, providing a more robust platform for drug testing. Using this approach, we identified HDAC inhibitors as active across multiple UPS and LMS models. Although the screen was limited by the use of single biological replicates, standard high-throughput screening quality control metrics ensured assay robustness, and follow-up dose‒response validation confirmed the initial hits. Pan-HDAC inhibitors with a hydroxamic acid backbone showed strong activity, with 11 out of the 12 compounds in the top 50% included in the screen. Class I-selective inhibitors (or dual-class I/II inhibitors) demonstrated high activity, with 9 out of 10 compounds ranking within the top 50%. Conversely, two class IIa-selective inhibitors showed limited activity and were not among the top-performing compounds. Quisinostat possesses subnanomolar specificity for class I HDAC isoforms, particularly HDAC1 and HDAC2. It is classified as a second-generation HDAC inhibitor, which was designed for improved *in vivo* efficacy in solid malignancies and shows superior HDAC inhibition to other HDAC inhibitors, including vorinostat and panobinostat.^36^ Quisinostat exhibited low nanomolar activity across all cell lines, and although this is the first report on the activity of quisinostat in UPS and LMS, the activity of other HDAC inhibitors across a range of STS subtypes has been reported.^37^ Mechanistically, HDAC inhibitors are known to induce cell cycle arrest, as HDAC1 represses the cyclin-dependent kinase inhibitor p21.^32^ Consistent with increases in p21 in other STS subtypes following HDAC inhibition,^37^there was a strong increase in p21 following quisinostat treatment. We also observed evidence of synergy between quisinostat and doxorubicin with increased apoptosis and the induction of DNA damage, which may be linked to increased DNA accessibility and the downregulation of DNA damage repair genes, as has been shown with other HDAC inhibitors.^38^^,^^39^ Additionally, in rhabdomyosarcoma, synergy with doxorubicin was associated with increased apoptosis and a change in the ratio of pro- and antiapoptotic proteins.^40^ We also showed anti-tumor activity *in vivo* with combined quisinostat and doxorubicin treatment, which resulted in a greater reduction in tumor volume and increased DNA damage compared to either single agent treatment. Further studies are now needed to confirm this in additional models and increased sample sizes. Interestingly, *in vivo* studies have shown that quisinostat has increased bioavailability and prolonged elevation of p21 in an ovarian xenograft model compared to other HDAC inhibitors.^36^^,^^41^

We identified a number of genes previously linked with proliferation and apoptosis in STS that were altered in response to quisinostat. This included the downregulation of FOS-like antigen 1 *(FOSL1*), which has been associated with the potent anti-tumor effects of panobinostat in UPS.^16^ We also observed an increase in *EPAS1,* which encodes HIF2α, following quisinostat treatment. Vorinostat has been shown to restore *EPAS1* expression in a genetically engineered mouse model of UPS, which leads to a reduction in tumor growth.^34^ Other studies on UPS have shown that the combination of vorinostat with a bromodomain-containing protein 4 (BRD4) inhibitor significantly promoted an unfolded protein response (UPR) and autophagy, as well as the induction of a muscle differentiation program. These effects are mediated via epigenetic silencing of the Hippo pathway transcriptional effector Yes-associated protein 1 (YAP1) inhibitor angiomotin (AMOT) and subsequent downregulation of Hippo/YAP1 signaling.^35^ We observed significant upregulation of *AMOT* after quisinostat treatment. Additionally, quisinostat increased *FOXO1* expression*.* Activation of *FOXO1* transcriptional activity in synovial sarcoma promotes apoptosis and autophagy, suggesting a potential role for *FOXO1* in mediating the therapeutic response to quisinostat.^33^ The same study carried out RNASeq analysis across a panel of synovial sarcoma cell lines treated with quisinostat and showed changes in genes associated with cell-cycle arrest, neuronal differentiation, response to oxygen-containing species and apoptosis. This finding correlated significantly with differentially expressed gene sets across different tumor types following treatment with other HDAC inhibitors, suggesting that there may be a core HDAC inhibitor-regulated gene set.^33^ Thus, several genes that are altered by quisinostat are linked to potential anti-tumor effects in STS and further mechanistic studies are needed to define their role in UPS and LMS.

Despite evidence supporting a role for HDACs in driving a more malignant phenotype in sarcomas and encouraging pre-clinical studies, clinical trials with HDAC inhibitors in STS have been disappointing overall. However, these previous studies of HDAC inhibitors in patients with sarcoma included heavily pre-treated patients with multiple different sarcoma histotypes.^20-26^ Furthermore, the majority of these studies did not include translational sub-studies to investigate biomarkers of response,^20-24^^,^^26^ and many were investigating HDAC inhibitors as a monotherapy.^20^^,^^23^^,^^24^ Despite these disappointing results in unselected STS populations, there were reports of disease stability and partial or complete responses in patients with both UPS and LMS.^20^^,^^21^^,^^24^^,^^26^^,^^42^ The numbers, however, are low, highlighting the need for biomarkers to guide patient selection. Predictive biomarker discovery is particularly challenging to investigate in rare tumor types, such as UPS and LMS. This could be explored using multi-omic analysis of clinically relevant patient-derived models,^43^ retrospective analysis of tumor samples treated with HDAC inhibitors in previous early-phase clinical trials, and translational analysis of both pre- and on-treatment tumor biopsies during a prospectively conducted HDAC inhibitor clinical trial.

Though investigation of response biomarkers to HDAC inhibitors in clinical samples has been limited, some interesting pre-clinical data investigating this topic exist. In certain instances, sensitivity to HDAC inhibitors correlates with HDAC overexpression or gene amplification, suggesting a potential role as biomarkers. For example, in a panel of liposarcoma cell lines, the expression of *HDAC1* correlated with sensitivity to quisinostat.^44^ However, this relationship is not universal, and HDACs as predictive biomarkers remain poorly defined. Indeed, we show here that although copy number loss is a frequent event in UPS and LMS, it was not sufficient to predict sensitivity to HDAC inhibitors in our panel of cell lines. Increasing the number of available models and extending the study to look at wider transcriptional changes will be needed to establish whether more specific markers, or a panel of markers, will be needed. Potential candidates include the UV excision repair protein RAD23 homolog B (RAD23B, also known as HR23B), which is involved in nucleotide excision repair mechanisms.^45^ In STS, a significant correlation was observed between baseline HR23B expression and response to vorinostat.^46^ In liposarcoma, *GYS1,* the gene encoding glycogen synthase, predicted response to quisinostat, while analysis of global gene changes demonstrated that pathways involved in DNA replication, mitosis, and cell cycle were enriched for biomarkers of poor response to quisinostat, suggesting that highly proliferative cells were more resistant.^44^

A more favorable pharmacokinetic profile and enhanced efficacy of quisinostat, resulting in lower doses required clinically, support the use of quisinostat over other HDAC inhibitors.^41^^,^^47^ It has been tested in early clinical trials across various cancers, both as a single agent and in combination with other agents.^47^^,^^48^ Our data support combining quisinostat with standard-of-care doxorubicin, with synergy observed across the UPS models. However, for clinical application, this will require further pharmacodynamic studies and rigorous toxicity testing. Though the combination of quisinostat with doxorubicin has not been assessed in early-phase clinical trials, multiple phase I/II trials of other HDAC inhibitors (abexinostat, panobinostat, belinostat) in combination with anthracyclines have demonstrated a tolerable safety profile, suggesting this combination has clinical potential.^22^^,^^25^^,^^26^ Further functional validation of the biomarkers associated with the response to quisinostat is needed to support moving this combination to the clinic. Additionally, the development of isoform-selective inhibitors, which offer the potential for greater biological precision and reduced toxicity associated with the use of HDAC inhibitors,^49-53^ may provide a more effective way forward.

## Materials and methods

### Cells and culture media

SHEF_UPS01, SHEF_UPS02, SHEF_UPS03 and SHEF_UPS04 cell lines,^54^^,^^55^ provided by Dr Karen Sisley (University of Sheffield), were maintained in RPMI-1640 (Gibco; #11875093) supplemented with 20% (v/v) fetal bovine serum (FBS) (Gibco; #10270106), 1% (v/v) antibiotic-antimycotic (Thermo Scientific; #15240062) and 0.4% (v/v) D-glucose (Sigma–Aldrich; #98644). IEC-56 cells were established at the CRUK Scotland Centre^8^ and maintained in Advanced DMEM (Gibco; #12491015), 10% (v/v) FBS, 1% (v/v) penicillin‒streptomycin (Gibco; #15140122), and 1% (v/v) GlutaMAX (Thermo Scientific; #35050061). SK-UT-1 and SK-LMS-1 cells were maintained in Minimum Essential Medium Eagle (Sigma–Aldrich; #M4526) supplemented with 10% (v/v) FBS, 1% (v/v) penicillin–streptomycin and 1% (v/v) L-glutamine (Gibco; #11539876). IEC005,^56^ provided by Dr David Moura and Prof Javier Martin-Broto, was maintained in RPMI-1640 supplemented with 10% (v/v) FBS and 1% (v/v) penicillin‒streptomycin. ICR-LMS-1^57^ and ICR-LMS-4 were maintained in DMEM F/12 (Gibco; #11320033), 10% (v/v) FBS, 1% (v/v) L-glutamine, 0.5% (v/v) penicillin‒streptomycin, 5.6 μM Y27632 (AOBIOUS; #AOB3877), 5 μg/mL human insulin solution (Sigma–Aldrich; #I9278), 400 ng/mL hydrocortisone (Stem Cell Technologies; #74142), 280 ng/mL amphotericin B (Gibco; #15290-026), 10 ng/mL EGF (Peprotech, #31509) and 9.62 ng/mL Cholera Toxin (Merck; #C8052). ICR-LMS-4 was generated as described previously.^57^ All the cell lines were incubated at 37 °C in a humidified incubator with a 5% CO_2_ atmosphere. The cell lines were routinely confirmed to be mycoplasma negative and authenticated by short tandem repeat profiling.

### Ethics and clinical samples

The study was conducted in accordance with the guidelines of general approval for the use of surgically obtained tissue, with approval (IRAS ID 306447) from NHS Research Scotland (NRS) Greater Glasgow and Clyde Biorepository (West of Scotland Research Ethics Committee 4, reference number 22/WS/0020). Written informed consent was obtained from all the individual participants included in the study, and all the procedures conformed to the principles of the Declaration of Helsinki.

An undifferentiated pleomorphic sarcoma tumor sample (EDI-UPS-1) was collected from the shoulder/axilla of a 77-year-old male, and a stage 4B high-grade uterine leiomyosarcoma tumor sample (EDI-LMS-1) was collected from a 47-year-old female who had undergone a total abdominal hysterectomy with bilateral salpingo-oophorectomy. For both samples, the fresh tumor tissue was transported in MACS Tissue Storage Solution (Miltenyi Biotec; #130-100-008) before mechanical and enzymatic dissociation. In brief, the samples were minced (~1 mm^3^) and digested in Advanced DMEM supplemented with 5% (v/v) FBS, 1% (v/v) penicillin–streptomycin, 1% (v/v) insulin-transferrin-selenium (Gibco; #41400-045), 0.5  mg/mL collagenase (Sigma–Aldrich; #C9891), 0.1 mg/mL hyaluronidase (Sigma–Aldrich; #H3506), 0.1 mg/mL DNase I (Sigma–Aldrich; #10104159001), 10 ng/mL EGF and 10 μg/mL hydrocortisone for 1 h at 37 °C, with agitation. After digestion, the suspension was incubated in RBC lysis buffer (Invitrogen), followed by incubation with 0.05% trypsin/EDTA and 1 mg/mL DNase I before being passed through a 70 μm filter. All reagents were supplemented with 10 μM Y27632 (AOBIOUS; #AOB3877). The cells were expanded in culture before being seeded for compound testing in DMEM F/12 supplemented with 10% (v/v) FBS, 1% (v/v) L-glutamine, 0.5% (v/v) penicillin–streptomycin, 5.6  μM Y27632, 5  μg/mL human insulin solution, 400 ng/mL hydrocortisone, 280 ng/mL amphotericin B, 10 ng/mL EGF, and 9.62 ng/mL cholera toxin. This project is covered by NHS Research Scotland (NRS) Greater Glasgow and Clyde Biorepository ethics approval, 22/WS/0020.

### Drug screens

High-throughput screening was carried out in barcoded 384-well imaging plates (Greiner Bio-One; #781091). The cells were seeded and incubated for 24 h before 72-h drug treatment. The Prestwick FDA Approved Chemical Library (1280 compounds; Prestwick Chemical) and SCREEN-WELL PKE library (176 compounds; Enzo Life Sciences) were tested in a single replicate at 10 µM on 4 UPS cell lines (SHEF_UPS01, SHEF_UPS02, SHEF_UPS03 and SHEF_UPS04). The custom HDAC inhibitor library (53 compounds, Supplementary Table S1) was tested in a single replicate across an 8-point half-log dose‒response (10−0.003 µM) on all UPS and LMS cell lines. All plates included negative (0.1% v/v DMSO *n* = 64) and positive (1 μM staurosporine *n* = 64) controls. The plating and distribution of the drug library source plate(s) were randomized, with the exception of the well location of the negative and positive controls. After treatment, the cells were fixed with 4% formaldehyde (Sigma–Aldrich; #252549) and stained with 4 µM Hoechst 33342 (Thermo Scientific; #62249) for 30 min. Four fields of view were captured using a 10× objective on an ImageXpress® Micro XL widefield high-content microscope (Molecular Devices). For the Prestwick Chemical and SCREEN-WELL PKE libraries, the MetaXpress (Molecular Devices) built-in analysis modules were used to automate the counting of nuclei (with user-defined thresholds) and the cell cycle stage (determined by measuring the integrated and average intensity values of the DNA content from the DAPI channel using the nuclear mask). The cell and image data were exported as well-level median values and analyzed using Spotfire High Content Profiler software (Revvity Signals). Briefly, the data were median normalized to plate controls, followed by median polish intraplate normalization. The data were subsequently transformed into a principal component space. The Euclidean distance (phenotypic distance) was calculated relative to the centroid of the DMSO controls. A phenotypic distance with a z-score greater than 3 was considered a hit compound. For the HDAC inhibitor library, the MetaXpress (Molecular Devices) built-in analysis modules were used to automate the counting of nuclei (with user-defined thresholds). The image data were exported as total values across sites. Total nuclei counts (sum nuclei counts over 4 sites) were median normalized to that of the DMSO vehicle controls on a plate-by-plate basis to determine the percentage viability and z-score quantification. Robust Z prime signal-to-noise analysis was good, ranging from 0.58 to 0.79 for 17/18 plates; the robust Z prime for SK-LMS-1 (Plate 1) was poor (−0.001); as such, this plate was excluded from hit selection. Hits were defined as having a z-score less than −4 in at least 3 cell lines at 0.3 µM.

### Hit validation

Hit compounds (Supplementary Table S2) were validated in an 8-point half-log dose‒response, and the highest dose was adjusted as required for potent compounds. The cells were seeded into 384-well plates and incubated for 24 h, followed by compound addition and incubation for 72 h. All plates included negative (0.1% v/v DMSO) and positive (1 μM staurosporine) controls for normalization. End-point staining was carried out as described above, with the percentage viability determined from the total nuclei counts (sum nuclei counts over 4 sites) median normalized to that of the DMSO vehicle controls on a plate-by-plate basis. Four-parameter log-logistic dose‒response models were used to calculate IC_50_ values (nonlinear regression curve fitting), and area under the curve analyses using GraphPad Prism 10 (v10.4.1). Compounds with an IC_50_ value less than 1  µM across at least 3 cell lines passed validation.

### Combination studies

The four best-performing single agents were tested in a 6 (doxorubicin) × 5 (HDAC inhibitor) dose‒response matrix across all cell lines. After 72 h of treatment, percentage viability was determined using nuclei counts and analyzed with synergyfinderplus.org^58^ using the ZIP, HSA, Bliss and Loewe synergy models following the threshold definitions established by Ianevski.^31^

### Apoptosis

Cells were seeded into 384-well plates and incubated for 24 h before the addition of 0.1% DMSO, 10 nM quisinostat, 100 nM doxorubicin or a combination of the two, and NucView® Caspase-3 Enzyme Substrates (Biotium; #10403). The wells were imaged using the IncuCyte S5 Live-Cell Analysis System (Satorius) for 72 h. Phase-contrast and green fluorescent images were acquired every 3 hours and analyzed using the built-in basic analysis software.

### Western blotting

The cells were lysed in cold RIPA buffer (Thermo-Scientific; #89900) supplemented with PhosSTOP™ Phosphatase Inhibitor (Roche; #4906845001) and cOmplete™ ULTRA Protease Inhibitor (Roche; #5892953001). Proteins were resolved in 4-15% Mini-PROTEAN® TGX gels (Bio-Rad; #4568084) and transferred onto PVDF membranes (Bio-Rad; #1704157). The membranes were blocked in 5% (w/v) BSA (Sigma–Aldrich; #12659) in TBS-Tween 20 (TBS-T; Sigma–Aldrich; #P1379) followed by incubation at 4 °C overnight in primary antibodies (Supplementary Table S3) diluted in blocking buffer. The membranes were washed three times in TBS-T and incubated with 1:10000 HRP-conjugated anti-mouse (Cell Signaling Technology; #7076) or anti-rabbit (Cell Signaling Technology; #7074) secondary antibodies for 1 h. The washes were repeated, and the membranes were visualized with Clarity Western ECL Substrate (Bio-Rad; #1705061) on an ImageQuantTM 800 (Cytiva).

### Quantitative PCR

The cells were washed once with PBS, and RNA was extracted using the RNeasy Kit (Qiagen) according to the manufacturer’s instructions. The RNA quality and concentration were assessed using the NanoDrop 2000c spectrophotometer (Thermo Fisher; #ND-2000). cDNA was synthesized from 1 µg of total RNA using the SuperScript First-Strand Synthesis System for RT-PCR (Invitrogen). qPCR was performed using 10 ng cDNA, gene-specific primers (IDT; sequences in Supplementary Table 4), and SYBR Select Master Mix (Applied Biosystems; #4472908) for 40 cycles. The data were collected on the QuantStudio 3 Real-Time PCR System (Applied Biosystems) and analyzed using the accompanying software. Gene expression was quantified using the ΔΔCt method and normalized to RPS13 expression.

### Mouse studies

All mouse studies were performed in accordance with UK Home Office regulations, under project license PP7510272, and were approved by the Animal Welfare and Ethical Review Board of the University of Edinburgh (PL05-21). Female Rag2-Il2rg double knockout (R2G2) mice (Inotiv), aged 6–8 weeks, were housed in individually ventilated cages (Techniplast Blue Line 1284L) under standard conditions (temperature: 20–24 °C; humidity: 45%–65%) with a 12-h light/dark cycle. Bedding (Datasand Eco Pure Aspen Chip ECO2, Datasand Paper Shavings RDJ91 (S)) and environmental enrichment (Datasand Aspen Bricks, Dome Homes, and Polycarbonate Tunnels) were provided. The mice had ad libitum access to an SDS RM3 (P)-25 VP diet (DS801190G10R). The mice were permitted to acclimatize for a minimum of 1 week prior to any intervention.

Each mouse (*n* = 20 total) was considered an experimental unit. The mice were bilaterally implanted subcutaneously with 2.5 × 10^6^ IEC-56 cells in 100 µL of PBS. When the tumors reached 40−60 mm^3^, the mice were randomized into four treatment groups (*n* = 5 per group). Tumors were allocated to treatment groups using volume-based equalization to minimize initial group imbalances.

Mice were treated for 14 d by intraperitoneal injection (0.1 mL/20 g): (1) vehicle (daily, maximum 0.01% DMSO in ultrapure water), (2) doxorubicin (weekly, 3 mg/kg in ultrapure water), (3) quisinostat (daily, 3 mg/kg in maximum 0.01% DMSO in ultrapure water), or (4) a combination of doxorubicin and quisinostat. The mice were monitored daily during treatment and weighed biweekly. Tumors were measured biweekly with calipers, and volume was calculated with the formula: volume = 0.5 × length × width^2^. To reduce potential confounders, the animals were housed in the same cage position throughout treatment, treated at consistent times each day, and handled by the same personnel using non-aversive methods. Blinding was not performed, although the individuals administering treatment and taking measurements were unaware of the expected outcomes of the study.

The mice were culled in accordance with UK Home Office regulations by cervical dislocation, with cessation of circulation (severing a blood vessel) as the confirmation method, 24 h after the final treatment, or when they exhibited clinical signs of ill health. On cull, tumors were excised, weighed, and stored in 10% neutral buffered formalin for embedding.

### Immunohistochemistry

Formalin-fixed paraffin-embedded (FFPE) tumor sections were stained as described previously, following incubation at 4 °C overnight in primary antibodies (Supplementary Table S3).^8^ The sections were imaged using the NanoZoomer slide scanner (Hamamatsu) and analyzed on QuPath (v0.5.1).^59^

### Genomic analysis

The samples used in this genomic analysis include Pan Cancer Analysis of Whole Genomes (PCAWG)^30^ LMS patient samples (*n* = 15), Genomics England (GEL)^60^ LMS (*n* = 84) and UPS (*n* = 32) patient samples, Edinburgh UPS solid tumors (*n* = 20) and UPS cell lines previously sequenced by our group (*n* = 4).^8^ For more information, see Supplementary Information.

### Statistical analysis

All experiments were repeated at least two times; full details of biological and technical replicates are provided in the corresponding figure legends. The experimental data are expressed as the means  ±  standard deviations, and analyses were performed in GraphPad Prism 10 (v10.4.1). IC_50_ values and AUC were calculated from four-parameter log-logistic dose‒response models. The difference between multiple groups were analyzed using a one- or two-way ANOVA with Tukey’s multiple comparison tests. *p*-values less than 0.05 were significant, *p < 0.05, **p < 0.01, ***p < 0.001, ****p < 0.0001.

## Data Availability

The datasets generated and/or analyzed during this study can be accessed via the International Cancer Genome Consortium (ICGC) data portal (https://dcc.icgc.org/), the National Genomic Research Library v5.1 (https://doi.org/10.6084/m9.figshare.4530893.v7), and the raw screening data are available upon reasonable request.
